# Stripe Noise Removal in Blazed Grating Generation for Electrically Tunable Beam Deflector

**DOI:** 10.3390/ma18020291

**Published:** 2025-01-10

**Authors:** Woosup Kim, Taeyoung Kim, Jun Do, Heechang Ma, Heesun Yoon, Kanghee Won

**Affiliations:** 1 Department of Information Display, Kyung Hee University, Seoul 02447, Republic of Korea; kws2543@khu.ac.kr (W.K.); kimtaeyoung865@khu.ac.kr (T.K.); juntao@khu.ac.kr (J.D.); decemach12@khu.ac.kr (H.M.); 2 SOS LAB Co., Ltd., 303, Research Institute of University-Industry Cooperation Center, GIST 123 Cheomdangwagi-ro, Buk-gu, Gwangju 61005, Republic of Korea; hyoon@soslab.co

**Keywords:** liquid-crystal device, blazed phase grating, beam deflector, phase wrapping, stripe noise, phase modulation

## Abstract

In this paper, we demonstrate a blazed phase grating to achieve tunable beam steering and propose a novel algorithm to reduce the stripe noise in wrapped phase. To control the diffraction angle to steer light to the desired direction, an electrically tunable transmission-type beam deflector based on liquid crystals is introduced, and electric fields are applied to the patterned indium tin oxide electrodes to change its phase retardation. Two different 2π phase-wrapping methods are applied to obtain various diffraction angles within the minimum cell-gap, and the method of equal interval of phase achieves a worthwhile diffraction efficiency compared to the methods based on equal interval of diffraction angle. The proposed method is able to completely eliminate the stripe noise in all steering angles that helps to improve the diffraction efficiency.

## 1. Introduction

Stripe noise is a common noise observed in optical sensors and electronic systems. The causes of stripe noise are multifaceted and can be broadly categorized into device-related factors, optical influences, and the effects of applied code. From a device perspective, stripe noise can arise from the scanning response of sensors [1], variations in pixel responses within the sensor array [2], imbalances in the response of image detection systems [3], and differences in characteristics between devices [4,5,6,7]. Optical influences include noise caused by unstable thermal properties of photoelectric systems [8] and optical interference [9].

This paper focuses on stripe noise in optical device systems where liquid crystals (LCs) are electrically controlled by the applied optical phase profile, enabling light to be steered in the desired direction and proposes a solution to mitigate this issue. Optical device systems that steer light in the desired direction have been researched and developed for various applications, including mechanical and non-mechanical types [10,11,12,13,14,15,16]. Compared to large mechanical beam-steering systems, non-mechanical systems offer several advantages, such as a smaller and more compact design, lighter weight, enhanced reliability, and lower power consumption. Recently, the use of nano-material-based metamaterials for steering light has also been actively studied [17,18,19]. Among them, the electrically tunable beam deflector (BD), which is a non-mechanical type of beam-steering system using LC [20,21,22,23,24,25,26,27] utilizing LC, steers the incident beam to a desired angle by modulating the refractive index of the LCs to create a precise phase profile. In addition to the method we proposed, other technologies can achieve phase modulation by calculating and adjusting phase values using digital control. These methods include modular operation-based wrapping (which automatically corrects over- or under-shoot values), loop control systems (which dynamically adjust the voltage input to the LC cell in real time), and table-based control (which employs a look-up-table to store the relationship between voltage and phase output for precise modulation) [28]. This paper demonstrates how precise phase profiles can be implemented through LC to steer light to the desired angle.

**Figure 1:**
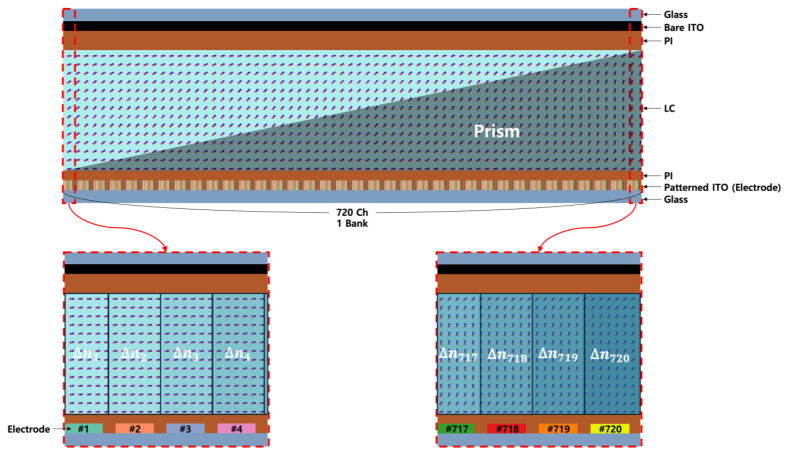
Assigning materials to a BD. The TechWiz LCD 2D simulator (Sanayi, Incheon, Korea) was utilized. The voltage applied to each channel tilts the LC, causing a change in ∆n, which in turn leads to phase retardation.

In order to achieve various diffraction angles, the optical phase profile, which is formed according to the birefringence of the LC, must be changed in real time. For this, various voltages must be applied to the individual electrodes on the bottom of the BD and a driving module with 720 channels is designed to independently operate each patterned electrode. Figure 1 depicts the structure of a BD with indium tin oxide (ITO) electrodes and LC for the birefringence changes [29]. The minimum measurement width, critical dimension, can be reduced to 0.5 µm by the optimization in the stepper process, and the patterned ITO electrodes on the lower substrate, having a width of 1.5 µm with a space of 0.5 µm, were fabricated. Different voltages are applied to each electrode, which alters the alignment angle of the LC. The degree of tilting in the LC changes its birefringence. The birefringence (∆*n*) is determined as the difference between the effective refractive index $n_{eff}\left(\theta \right)$) of the LC at a specific tilt angle (*θ*) and the ordinary refractive index ($n_{o}$). The effective refractive index ($n_{eff}\left(\theta \right)$) depends on the orientation of the LC molecules relative to the applied electric field and is described by the following Equation:

(1) $$n_{eff}\left(\theta \right)=\frac{n_{o}n_{e}\;}{\sqrt{{n_{e}}^{2}{sin}^{2}\theta +{n_{o}}^{2}{cos}^{2}\theta \;}}$$

where $n_{e}$ is the extraordinary refractive index. The birefringence equation is given as follows:

(2) $$∆n\;={\;\;n}_{eff}\left(\theta \right)-n_{o}$$

The birefringence is maximized θ=0°, meaning no external voltage is applied, and minimized when θ=90°, corresponding to the maximum applied voltage.

**Figure 2:**
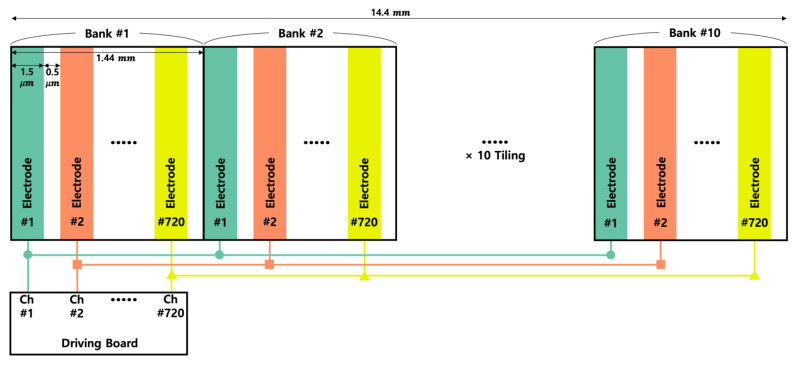
The concept of tiling process.

The BD has been introduced for compact system and researched and developed for and eye-tracking system in holographic display [30]; however, there is a need for the development of devices with larger viewing angles. For this reason, the patterned ITO with a smaller pitch must be implemented by the use of a reduction stepper (step and repeat projector) process [21,31,32,33]; however, while the pitch decreases, the total size of the active area is decreased due to the limited number of channels. This can be improved by the introduction of a periodic tiling process, and the active area is expanded by repeating the unit bank [29]. When a total of 720 electrodes are grouped into a unit bank and repeated 10 times, the active area of only 1.44 mm at a 2 µm pitch patterns can be increased to 14.4 mm, as shown in Figure 2. If a specific voltage is applied to the channel #1, the same voltages are transferred to each channel #1 of 10 unit banks since the same channels of each unit bank are connected to each other through the via-hole process. Similarly, the same voltage is transmitted to channel #2 of all 10 unit banks. In this way, the same voltage is transmitted to the electrodes with the same sequence number across all 10 unit banks.

There are various errors that may occur during the fabrication of micro-patterns on the lower substrate and LC cells. LC can exhibit differences in uniformity due to LC material non-uniformity (difference in Δ*n*) or fabrication error (cell gap, spacer, electrode pixel pitch). The detailed process for fabricating the micro-patterning for the lower substrate and LC cell is provided in Supplementary Note S1.

**Figure 3:**
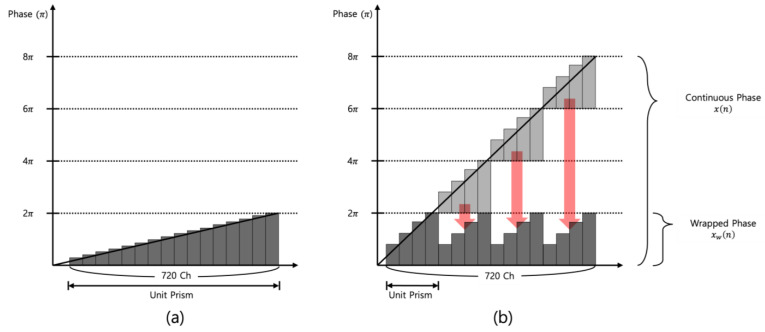
The concept of continuous phase and wrapped phase. (**a**) represents the case where the unit prism is 1, and (**b**) represents the case where the unit prism is 4. Furthermore, the red arrow illustrates the concept of a phase exceeding 2π being wrapped down to within the 0~2π range.

The steering angle, *θ*, can be expressed by following Equation:

(3) $$\theta =arcsin\left(\frac{\lambda }{N\times p}\right)$$

where *λ* is the wavelength of the light, the pixel pitch is *p*, and *N* is the total channel number for one unit prism as follows:

(4) $$N=\;\frac{m}{i}$$

where m is the total channel number, 720, which is determined by the driver IC in the driving module, and *i* is defined as total number of unit prism. Since at least 2 and the maximum of 720 channels are required to make a unit prism, the range of *i* is set from 1 to 360. When *i* is the minimum value of 1, linearly increasing voltages are applied between the minimum voltage of channel #1 and the maximum voltage of channel #720 for making one unit prism with 2π phase modulation. This represents the minimum steering angle $\theta_{min}$ of 0.021° at the wavelength of 532 nm. For *i* > 2, the phase-wrapping method is required to express the actual phase exceeding 2π since it can be expressed by dividing it into several unit prisms having a 2π phase modulation as shown in Figure 3 [29].

Figure 3b shows the number of unit prisms when the number of unit prisms is 4. The total phase signal calculated by applying arctangent function typically exceeds the range [0, 2π]; however, it can be kept within that range through the wrapped phase method. Assuming that *x*(*n*) is the original continuous phase signal over the range [0, 2π], the wrapped-phase signal $x_{w}(n)$ has the same value as *x*(*n*) and can be considered down to the range [0, 2π] since the phase jumps every 2π eventually repeat from the same starting point. This results in expressing the total phase modulation of tens of π by repeating a 2π wrapped phases [34,35,36,37]. The maximum phase modulation is required to obtain the maximum diffraction angle; it can be obtained by applying the minimum and the maximum voltage to each of the two adjacent electrodes and repeating it 360 times. This is the same as the total maximum phase of 720π, as shown in the continuous phase. By applying an optical phase profile, a beam with a wavelength of 532 nm can be steered from 0° to a maximum of 7.643° in increments of 0.021°. There are several main factors that cause a decrease in diffraction efficiency (DE) when the beam is steered. The first factor is the fringe field effect, where DE decreases as the beam is steered to higher angles [29]. The second factor is related to the optical phase profile approach, which can also contribute to efficiency loss. Traditionally, a uniform angular approach has been used to apply optical phase profiles for beam steering. However, this paper proposes a method to reduce stripe noise one of the main causes of efficiency degradation by generating and applying optical phase profiles using a phase modulation approach. This method improves DE by up to 21% [29].

## 2. Materials and Methods

As mentioned earlier, the maximum phase modulation that can be made with 720 channels is 720π, and this makes the maximum diffraction angle of 7.643° when the pixel pitch is 2 µm at the wavelength of 532 nm. In order to steer the light to desired direction, two different approaches have been explored: the equal interval of diffraction angle and the equal interval of phase.

**Figure 4:**
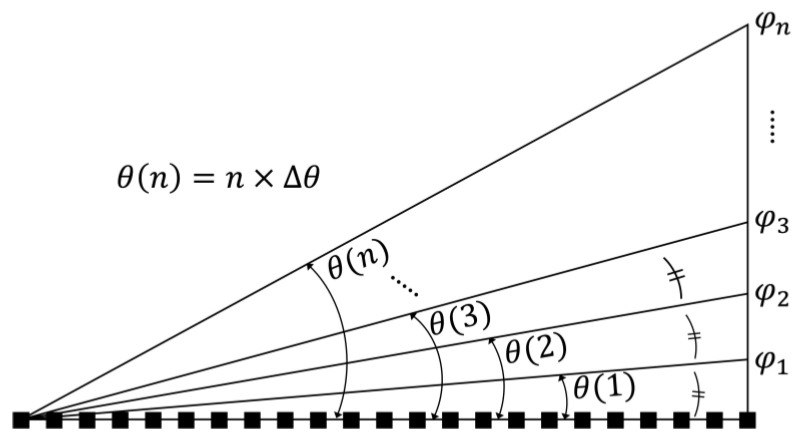
Conceptual diagram of equal interval of diffraction angle.

First of all, a method of uniformly dividing the maximum diffraction angle by 360 was applied, and the methodology for obtaining the linear diffraction angle is shown in Figure 4.

The maximum diffraction angle of 7.643° divided by 360 is 0.021°, and it is defined as the unit value of diffraction angle (∆*θ*). A specific diffraction angle reflecting a multiple number (*n*) of 1 to 360 in ∆*θ* can be defined as in Equation (5) below:

(5) $$\theta \left(n\right)=n\;\times \;\Delta \theta \;=\;\arcsin\left(\frac{\;\lambda }{\left(\frac{720}{i}\right)\times p}\right),\;0<n\leq 360$$

where *λ* is the wavelength of the light, the pixel pitch is *p*, and *i* is defined as total number of unit prism.

At a specific diffraction angle *n* × ∆*θ*, the value of *i* can be derived by substituting the fixed values of 532 nm and 2 µm for *λ* and *p*, respectively. The values of *i* are not an integer, which means an incomplete unit prism is generated when 2π wrapping method is applied. Then, the phase values ($\varphi_{n})$ corresponding to the specific diffraction angles were calculated; however, it was found that phase values are rarely also an integer.

(6) $$\varphi_{n}=2\;\times \;i=2\;\times \;\frac{sin\theta (n)\times \;(720\;\times \;p)}{\lambda }$$

**Figure 5:**
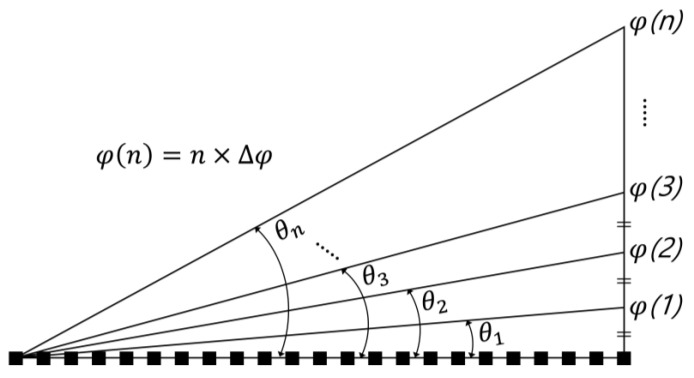
Conceptual diagram of equal interval of phase.

To create a complete unit prism by making phase values into integers, the maximum phase modulation was evenly divided into 360 steps. The methodology for applying this method to find the phase modulation is shown in Figure 5.

The maximum phase modulation (*φ*_max_) of 720π is equally divided by 360, and it is defined as the unit value of phase modulation (∆*φ*).

Then, the diffraction angle is calculated based on this Equation below:

(7) $$\theta_{n}=\arcsin\left(\frac{\;\lambda }{(2/\frac{\varphi (n)}{\varphi max})\times p}\right)=\arcsin\left(\frac{\;\lambda }{(\frac{720}{i})\times p}\right)$$

This method calculated the diffraction angle of $\theta_{n}$, corresponding to the phase retardation of *φ*(*n*), which is predefined by integer multiples. As shown in Equation (7), *i* becomes equal to the value of *n*, defined as an integer from 1 to 360. For this reason, no phase remnant occurs since *φ*(*n*) increases in multiples of 2π.

**Figure 6:**
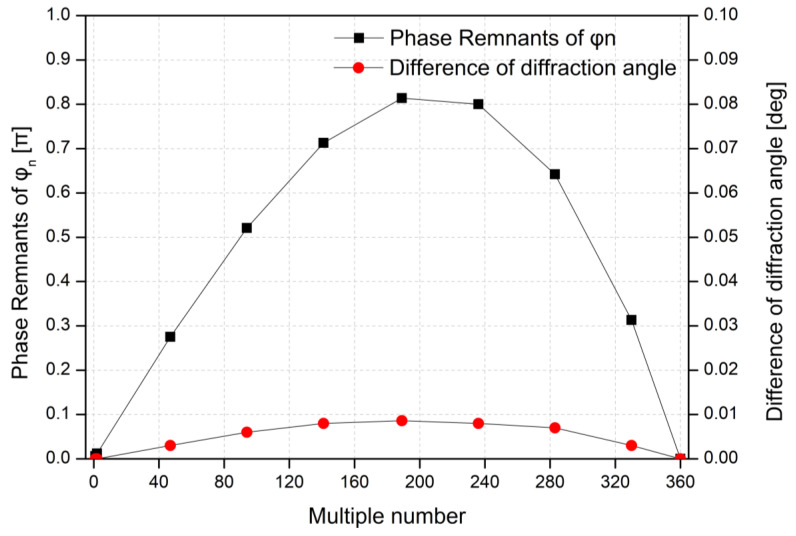
Phase remnants of $\varphi_{n}$ and difference in diffraction angles between *θ*(*n*) and $\theta_{n}$ as a function of multiple number at the wavelength of 532 nm.

Table 1, Table 2 and Table 3 show a numerical comparison of two methods, equal interval of diffraction angle and phase, for representative multiple numbers. Table 1 represents the results for the red wavelength (645 nm), Table 2 for the green wavelength (532 nm), and Table 3 for the blue wavelength (470 nm). In the case of an equal interval of diffraction angle, the remaining value of the decimal point for $\varphi_{n}\;$ always appears, and it almost reaches 0.8π when the multiple number is 200 at the wavelength of 532 nm. On the other hand, in the case of equal interval of phase, it is found that there are negligible differences between *θ*(*n*) and $\theta_{n}$, maintaining the value of *φ*(*n*) as a multiple of 2. The analysis of multiple numbers from 1 to 360 is as follows:

Figure 6 shows the decimal point remnants of $\varphi_{n}$ for multiple numbers, 0 to 360, at the wavelength of 532 nm. The phase remnants of $\varphi_{n}$ are defined as the decimal part of its value. This value continues to increase until the multiple number is around 200 and reaches the maximum value of about 0.81π. This has a significant impact on a stripe noise since the size of the incomplete prism is about 40% in terms of 2π wrapping. The difference between the diffraction angle of *θ*(*n*) and $\theta_{n}$ also reaches a maximum of 0.0087°, which corresponds to 0.15 mm from side to side at a viewing distance of 1 m [38]. This is a negligible difference because this is a value that does not deviate from a person’s pupil size [39,40].

**Figure 7:**
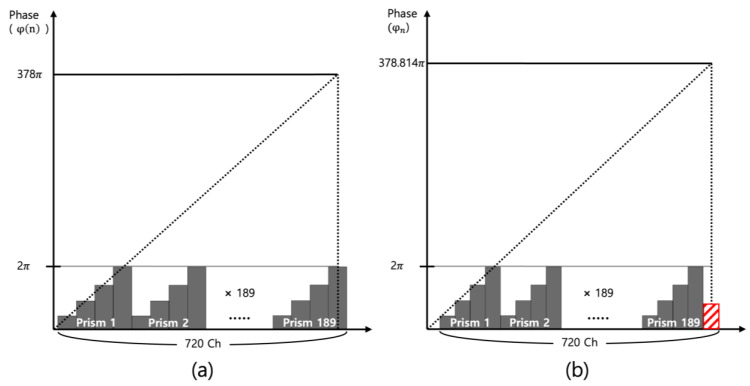
Phase-wrapping methods of (**a**) equal interval of phase and (**b**) equal interval of diffraction angle when multiple number (*n*) is 189.

As mentioned in Figure 2, a unit bank is repeated 10 times to increase the incidence area, and there is no problem when the number of phase-wrapped unit prism is formed to fit within 720 channels, as shown in Figure 7a. On the contrary, the phase-wrapped unit prism is not divided by an integer and remains as red as shown in Figure 7b. This is the main reason of the stripe noise when the phase remnant is repeated 10 times.

## 3. Results and Discussion

**Figure 8:**
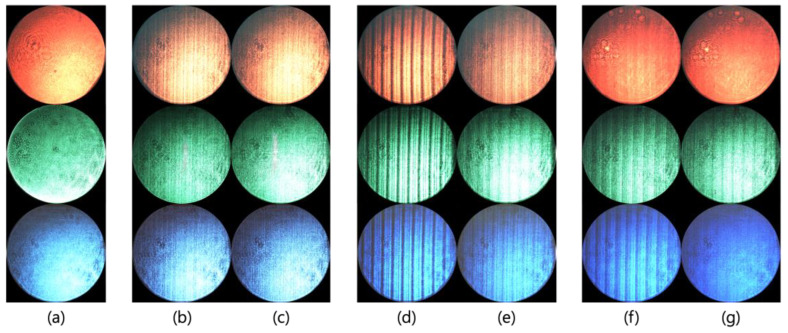
CCD images of diffraction patterns obtained experimentally for various multiple numbers (*n*). Images (**b**,**d**,**f**) represent the results from the equal interval of the diffraction angle method, while images (**c**,**e**,**g**) correspond to the equal interval of phase method. Image (**a**) shows the diffraction pattern at *n* = 0. Images (**b**,**c**) display the patterns at *n* = 47, (**d**,**e**) at *n* = 189, and (**f**,**g**) at *n* = 330. The CCD images are arranged in the order of red (645 nm), green (532 nm), and blue (470 nm) from top to bottom. Figure 8 includes only the CCD images for representative multiple numbers (*n*), but similar patterns are observed for values of *n* near these representatives.

An experiment was conducted to find out the difference between the equal interval of diffraction angle and phase. The laser at the wavelengths of 470 nm, 532 nm, and 645 nm is set up on the optical table, and the image of diffracted beam is captured by the charge-coupled device (CCD). Figure 8 shows the experimentally obtained diffraction images when multiple numbers (*n*) of 0, 47, 189, and 330 are applied to the cell. When the equal interval of the diffraction angle is applied, the stripe noise caused by the remaining phase increases around when *n* is 189, while it is clearly observed that the stripe noise decreases as *n* approaches either 47 or 330. This shows the same pattern as observed in Figure 6. We also evaluated stripe noise reduction using a signal-to-noise ratio (SNR) analysis, with ΔSNR representing the difference in SNR between the equal interval of diffraction angle method and phase method. Transitioning from the diffraction angle method to the phase modulation method significantly reduced noise and improved signal quality. ΔSNR values were minimal at multiple numbers, *n* = 47 and *n* = 330, where phase remnants were small, and higher at *n* = 189 due to larger phase remnants.

**Figure 9:**
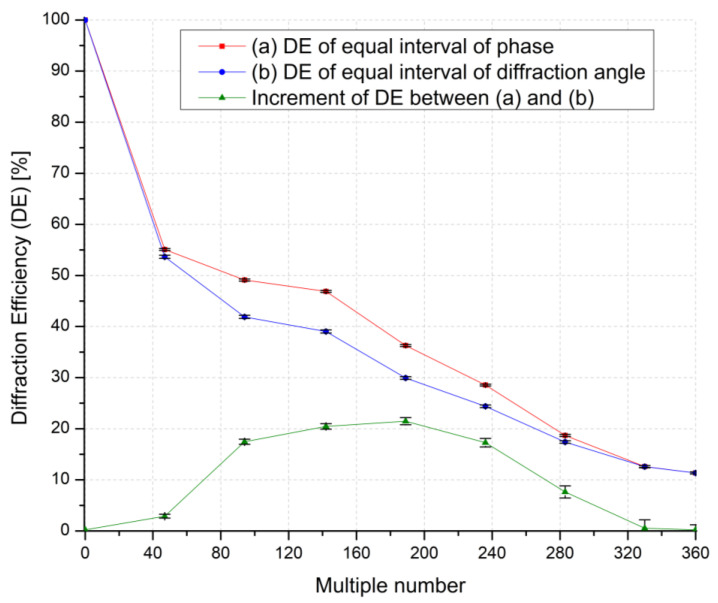
Experimentally measured DE of a LC BD as a function of the multiple number (*n*) and the difference between (a) equal interval of phase, (b) equal interval of diffraction angle, and the increment of DE between (a) and (b). The results of measuring a large number of DE were expressed as average values, and error bars were added based on the error data from these measurements.

The DE is measured to quantify stripe noise numerically since an increase in stripe noise leads to a loss of incident light, which results in a decrease in DE. As shown in Figure 9, the DE was measured for nine representative values within the multiple number (*n*) (0 to 360), and the values for two different methods were compared.

DE is defined as the ratio of $I_{str}/I_{off}$, where $I_{str}\;$ is the intensity of the steered beam, and $I_{off}$ is the maximum intensity of the light measured initially at 0° (when multiple number (*n*) is 0), where a linearly polarized beam from a laser at a wavelength of 532 nm is incident to the BD. The DE curves demonstrate that values start at 100% when the multiple number (*n*) is 0, and it gradually decreases as the multiple number increases. However, it was found that the DE of the equal interval of the diffraction angle is always lower than the DE of equal interval of phase, and the increment calculated as the ratio of their difference to the corresponding DE of equal interval of diffraction angle gradually increases and reaches the maximum of 21% at the multiple number 189. This result shows good agreement with the difference in the total phase modulation shown in Figure 6. Stripe noise intensifies as the phase remnants generated by applying the method of equal interval of diffraction angle increase, particularly as the multiple number approaches 189, leading to a reduction in DE.

## 4. Conclusions

In this paper, a BD with a 2 µm electrode pitch and 720 driving channels is proposed to steer the light at the maximum diffraction angle of 7.643° with angular resolution of 0.021° at the wavelength of 532 nm by repeating the unit prism of 2π using the phase-wrapping method. Initially, to accurately steer the beam to the desired angle, the diffraction angle of 7.643° was equally divided into 360 steps. And it was found that the method of equally dividing the diffraction angle generates the stripe noise in most cases. The stripe noise was quantified through the measurement of DE, and it was proven that the phase remnants generated by the equal interval of diffraction angle cause the stripe noise. The phase-wrapping algorithm for the equal interval of phase has been proposed to improve the stripe noise of the BD up to 21%. Although the equal interval of phase-wrapping algorithm slightly shifts the position of the transmitted image, the deviation is minimal, reaching only 0.15 mm at a 1 m distance. Considering that the human pupil is generally larger than 2 mm [39,40], this shift is negligible and does not affect practical performance. In order to remove the resulting stripe noises, we introduced the method for an equal interval of phase, and it was able to obtain clear images. We can expect that the improved imaging efficiency will open the way for applications including light detection and ranging (LiDAR), augmented reality and holographic displays, and optical communications where beam steering is applicable.

## Figures and Tables

**Table 1:** Calculation of diffraction angles and phase modulation for two different cases at red (645 nm).

Multiple Number (*n*)	Equal Interval of Diffraction Angle			Equal Interval of Phase		
	*θ*(*n*) [°]	$\varphi_{n}$ [π∙rad]	$\mathrm{Phase}\;\mathrm{Remnants}\;\mathrm{of}\;\varphi_{n}$ [π∙rad]	$\theta_{n}$ [°]	*φ*(*n*) [π∙rad]	Difference in Diffraction Angle [°]
1	0.026	2.009	0.009	0.026	2	0.000113
2	0.052	4.018	0.018	0.051	4	0.000225
47	1.211	94.405	0.405	1.206	94	0.005200
189	4.872	379.200	1.200	4.856	378	0.015457
330	8.506	660.462	0.462	8.500	660	0.005991
360	9.279	720.000	0.000	9.279	720	0.000000

**Table 2:** Calculation of diffraction angles and phase modulation for two different cases at green (532 nm).

Multiple Number (*n*)	Equal Interval of Diffraction Angle			Equal Interval of Phase		
	*θ*(*n*) [°]	$\varphi_{n}$ [π∙rad]	Phase Remnants of $\varphi_{n}$ [π∙rad]	$\theta_{n}$ [°]	*φ*(*n*) [π∙rad]	Difference in Diffraction Angle [°]
1	0.021	2.006	0.006	0.021	2	0.000063
2	0.042	4.012	0.012	0.042	4	0.000126
47	0.998	94.275	0.275	0.995	94	0.002907
189	4.013	378.814	0.814	4.004	378	0.008631
330	7.006	660.313	0.313	7.003	660	0.003338
360	7.643	720.000	0.000	7.643	720	0.000000

**Table 3:** Calculation of diffraction angles and phase modulation for two different cases at blue (470 nm).

Multiple Number (*n*)	Equal Interval of Diffraction Angle			Equal Interval of Phase		
	*θ*(*n*) [°]	$\varphi_{n}$ [π∙rad]	Phase Remnants of $\varphi_{n}$ [π∙rad]	$\theta_{n}$ [°]	*φ*(*n*) [π∙rad]	Difference in Diffraction Angle [°]
1	0.019	2.005	0.005	0.019	2	0.000043
2	0.037	4.009	0.009	0.037	4	0.000087
47	0.881	94.214	0.214	0.879	94	0.002001
189	3.543	378.634	0.634	3.537	378	0.005938
330	6.186	660.244	0.244	6.183	660	0.002294
360	6.748	720.000	0.000	6.748	720	0.000000

## Data Availability

The original contributions presented in this study are included in the article/Supplementary Materials. Further inquiries can be directed to the corresponding author.

## Supplementary Materials

The following supporting information can be downloaded at: https://www.mdpi.com/article/10.3390/ma18020291/s1, Note S1: Fabrication Process of Beam Deflector (BD).
