# Supplementary material for: Stripe Noise Removal in Blazed Grating Generation for Electrically Tunable Beam Deflector
Source: Materials (Basel). 2025 Jan 10;18(2):291. doi: 10.3390/ma18020291 (PMC11766839; doi:10.3390/ma18020291)
Supplement: Supplementary file 1 [file materials-18-00291-s001.zip › materials-3402429-supplementary.pdf]

Supplementary Note S1

[Fabrication Process of Beam Deflector (BD)]

The manufacturing process for BD is divided into the Micro-patterning fabrication for the lower substrate and the liquid crystal (LC) cell fabrication.

1. Micro-patterning fabrication for the lower substrate.

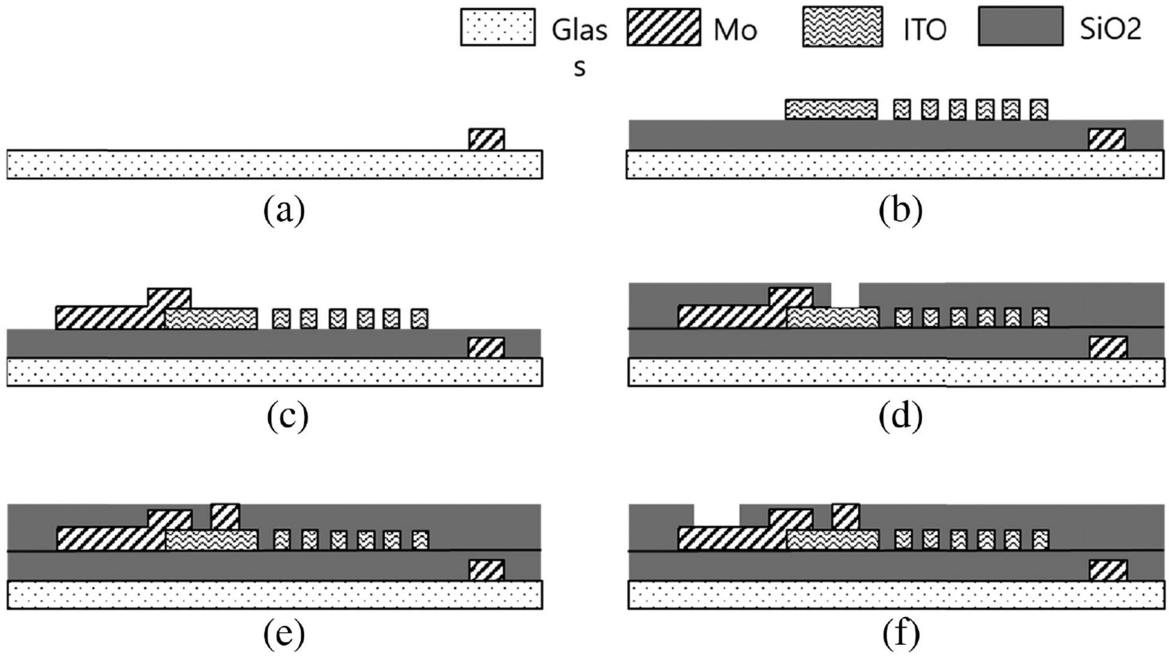

(a) Align key (Stepper)
(b) Signal line in the main area (Stepper)
(c) Signal line in the pad area (Aligner)
(d) VIA in the main area (Stepper)
(e) Contact in the main area (Stepper)
(f) Pad open in the I/O area (Aligner)

The lower substrate determines the maximum angle by the minimum pixel pitch of electrodes, and its fabrication process is as follows:

First, molybdenum (Mo) is deposited and etched to create an alignment key for precise mask

positioning during stepper (Nikon, Tokyo, Japan) lithography. Silicon dioxide, serving as a passivation layer, and indium tin oxide (ITO), for electrodes in the active area, are subsequently deposited onto the lower glass substrate using chemical vapor deposition and sputtering, respectively.

A mask with fine patterns for a 2 μm electrode pitch is applied to achieve a large steering angle. The stepper lithography's critical dimension is 0.5 μm, and the line space is eliminated through dry etching. In the mask aligner lithography step, a signal line for the pad is patterned to connect with the driving channels of the driver ICs.

The via-hole process is then developed and optimized to ensure proper connections for the 720 channels, maintaining the correct order for each bank. The via-hole size is set to 1 μm × 1 μm to account for process conditions and to ensure adequate resistance for the driver module's capabilities. The channels are aligned and connected using a contact process with Mo.

Finally, a pad opening process is performed to allow external signals to be applied through a flexible printed circuit board (FPCB). The chip-on-film driver ICs are bonded to the Mo pads on the lower glass substrate using anisotropic conductive film bonding.

2. LC cell fabrication.

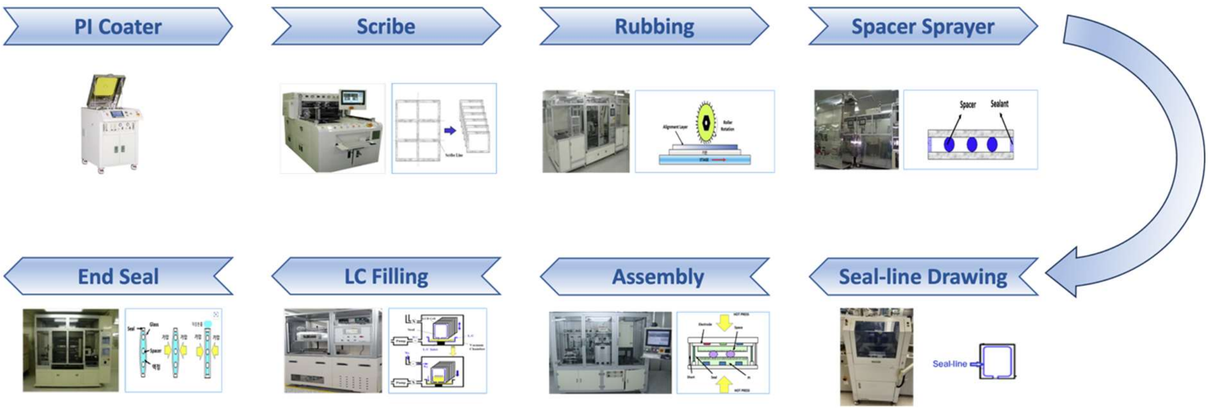

The following steps outline the process of making a LC cell featuring a micro-patterned ITO electrode on the lower substrate and a bare ITO electrode on the upper substrate. This process requires several key steps, starting from the spin coater to the end seal as follows:

a) Spin Coater (Midas system, Daejeon, Korea): This process is designed for thinly applying

polyimide (PI) onto wafers, achieving a maximum coating speed of 3000 rpm within 1 minute by utilizing multiple rpm steps.

b) Hot Chamber (Lisun, Kowloon, Hong Kong): A soft bake at around 120°C is carried out for several minutes to eliminate the solvent and stabilize the coating, followed by a hard bake at approximately 300°C for about an hour to complete the curing process.

c) Scriber (Shindo, Gyeonggi, Korea): To divide LC cells into the desired sizes from 8-inch circular wafers.

d) Rubbing (Shindo, Gyeonggi, Korea): Rubbing aligns the optical axis of the liquid crystal in a uniform direction. Since our BD operates as phase-only, the electrically controlled birefringence (ECB) mode is required. Therefore, the top and bottom plates were rubbed in the same direction.

e) Spacer Sprayer (Shindo, Gyeonggi, Korea): This maintains the cell gap of the liquid crystal between the top and bottom substrates. To achieve at least 2π phase modulation, we used liquid crystals with high birefringence (Δn=0.32) and 2.5 μm spacers to ensure a consistent cell gap.

f) Seal-line Drawing (Speedline, CA, USA): This defines the active area where the LC operates. The active area was set to 14.4 mm × 14.4 mm.

g) Assembly (Shindo, Gyeonggi, Korea): This is used to bond the upper and lower substrates together. The UV cure bond drawn in the seal-line drawing step was cured to maintain the final cell gap.

h) LC Filling (Shindo, Gyeonggi, Korea): This step injects the liquid crystal into the active area in a vacuum state.

i) End Seal (Shindo, Gyeonggi, Korea): This is the final step to seal the inlet of the active area where the liquid crystal was injected, based on the seal-line drawing.
